# A No-Code, Guideline-Based Custom GPT Outperforms Cardiologists in Response Quality for Cardiac Amyloidosis

**DOI:** 10.1016/j.jacadv.2026.102918

**Published:** 2026-07-22

**Authors:** Goro Fujiki, Satoshi Kodera, Hiroyuki Morita, Hideaki Morita, Norihiko Takeda

**Affiliations:** a Department of Cardiovascular Medicine, The University of Tokyo Hospital, Tokyo, Japan; b Third Department of Internal Medicine, Osaka Medical and Pharmaceutical University, Takatsuki, Japan; c International University of Health and Welfare, Tokyo, Japan

**Keywords:** artificial intelligence, cardiac amyloidosis, clinical decision support, GPT-4, large language model, practice guidelines

## Abstract

**Background:**

Cardiac amyloidosis (CA) is increasingly recognized in clinical practice. Whether a guideline-based large language model can deliver clinician-level answer quality for CA remains unknown.

**Objectives:**

This study aimed to develop a guideline-based custom generative pretrained transformer (GPT) (AmyloGPT) and evaluate whether its response quality matches or exceeds that of board-certified cardiologists for questions regarding CA.

**Methods:**

AmyloGPT was built in OpenAI’s GPT Builder without programming, integrating the 2020 Japanese Circulation Society CA guidelines as its knowledge base. Ten nonspecialist physicians generated 71 unique clinical questions. Five board-certified cardiologist answerers drafted responses. In a prospective, blinded, comparative study, evaluators (10 nonspecialists and 3 board-certified cardiologists) assessed paired responses for preference (forced-choice) and response quality using five-point Likert scales.

**Results:**

Compared with cardiologist answers, AmyloGPT was preferred in 81.1% (95% CI: 78.1%-83.8%) of evaluations by nonspecialist evaluators and 83.6% (95% CI: 78.6%-88.6%) of those by cardiologist evaluators (both *P* < 0.001). Among nonspecialists, AmyloGPT received higher median ratings for intent alignment and clinical usefulness (both *P* < 0.001). Among cardiologist evaluators, AmyloGPT received higher median ratings across all 5 quality dimensions: accuracy, consistency, validity, completeness, and absence of bias (all *P* < 0.001).

**Conclusions:**

A no-code, guideline-based custom GPT delivered superior response quality to that of cardiologists for CA questions. This approach allows clinicians without programming skills to build disease-specific large language models, potentially supporting equitable care where specialist access is limited. However, further studies are needed to evaluate potentially inaccurate outputs such as hallucinations.

Cardiac amyloidosis (CA), once considered rare, is now recognized as more prevalent than previously thought due to advances in noninvasive technetium-99m pyrophosphate scintigraphy.^1^^,^^2^ Among patients aged ≥65 years with preserved ejection fraction, 14.2% showed positive uptake on the scintigraphy,^3^ and 20% of those with heart failure and left ventricular hypertrophy had CA.^4^ With disease-modifying therapies such as tafamidis, early diagnosis has become directly linked to patient outcomes,^5^ highlighting the urgent need for pragmatic screening and diagnostic pathways.

Early detection of CA requires initial screening by nonspecialist physicians. However, CA symptoms are nonspecific and overlap with common cardiac disorders, making it crucial for general internists to consider CA at initial presentation. Yet nonspecialists often face challenges with key clinical decisions—which tests to order, how to interpret results, and when to refer—leading to diagnostic delays and missed diagnoses.^6^ Addressing these barriers requires accessible tools that enable timely access to specialized knowledge.

Large language models (LLMs), notably OpenAI’s GPT-4, have achieved approximately 80% accuracy and passing performance on national medical licensing examinations.^7^^,^^8^ They have also shown utility for clinical decision support.^9^ Guideline-based custom generative pretrained transformer (GPTs) integrating clinical practice guidelines as a knowledge base have demonstrated concordance rates of up to 87% with specialist recommendations.^10^ However, while general-purpose ChatGPT has been evaluated for amyloidosis,^11^ rigorous evaluations of disease-specific, question-answering LLMs designed to provide point-of-care decision support remain limited. Furthermore, artificial intelligence (AI) research in CA has mainly focused on diagnostic modalities such as electrocardiography, echocardiography, and scintigraphy,^12^^,^^13^ with limited work on LLM-based clinical decision support systems through interactive question-and-answering.

Accordingly, our primary objective was to determine whether a guideline-based custom GPT (AmyloGPT), integrating the 2020 Japanese Circulation Society (JCS) guidelines for CA as its knowledge base, could demonstrate response quality comparable to that of board-certified cardiologists for clinical questions posed by nonspecialist physicians. We developed AmyloGPT using GPT Builder (November 2023) without coding and conducted a prospective, blinded, head-to-head comparison of paired responses from AmyloGPT and cardiologist answers (Central Illustration). To the best of our knowledge, this is the first empirical evaluation of a guideline-based custom GPT against the clinical judgment of board-certified cardiologists in CA. If effective, this approach could support earlier recognition, timely referral, and appropriate management in routine practice, and may be generalizable to other rare-disease domains.

## Methods

This was a prospective, blinded comparative study evaluating response quality between a guideline-based custom GPT (AmyloGPT) and board-certified cardiologists. The study protocol prespecified primary outcomes, evaluation methods, and statistical analysis procedures prior to data collection.

### Participants and roles

We defined 3 mutually exclusive participant groups:

1) Question generators/nonspecialist evaluators (n = 10): physicians from internal medicine and related disciplines (including general internists, noncardiology specialists, and early-career physicians without board certification) with 3 to 14 years of clinical experience (mean ± SD, 7.1 ± 4.7 years; median, 4 years) drafted clinical questions regarding CA. These participants also served as nonspecialist evaluators, assessing response quality for both their own and others’ questions. Participant characteristics are detailed in Supplemental Table 1.
2) Cardiologist answerers (n = 5): five board-certified cardiologists with ≥10 years of clinical experience provided written responses. The total set of 71 questions was distributed among them, with each cardiologist responsible for 14 to 15 questions. They were permitted to consult guidelines, primary literature, and nongenerative online resources (eg, medical databases, professional society websites) but were explicitly prohibited from using generative AI tools (e.g., ChatGPT). Details are provided in Supplemental Table 1.
3) Cardiologist evaluators (n = 3): three board-certified cardiologists, independent of the cardiologist answerers, conducted blinded evaluations of response quality. Participant details are provided in Supplemental Table 1.

### Question development and deduplication

**Figure 1:**
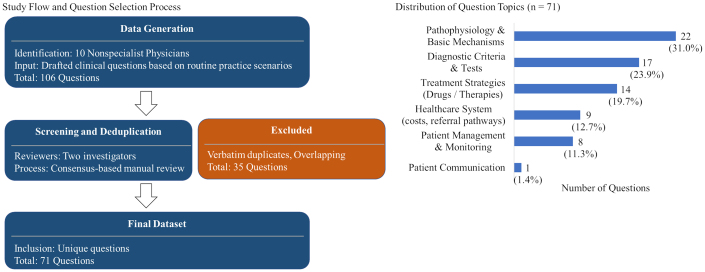
Selection Process of Clinical Questions for Analysis This flowchart illustrates the derivation of the final data set used for the comparative analysis. Initially, 106 free-text clinical questions regarding cardiac amyloidosis were generated by 10 nonspecialist physicians. Author-led deduplication was then performed by 2 investigators who jointly reviewed the submissions and, by consensus, removed verbatim duplicates and near-identical items. This process resulted in 71 unique clinical questions. The questions encompassed a broad spectrum of topics.

Ten nonspecialist physicians each generated approximately 10 open-ended, free-text questions regarding CA, reflecting diverse clinical uncertainties encountered in routine practice. Topics included diagnostic thresholds, test selection and interpretation, treatment options, patient communication, and health care system–related issues such as costs and referral pathways. This process yielded a total of 106 questions. Two investigators (G.F. and S.K.) reviewed the data set and, by consensus, removed verbatim duplicates and items with near-identical content, resulting in 71 unique questions (Figure 1). In cases where questions addressed similar topics but differed in specificity or phrasing, the version judged to be clearer and more generalizable to nonspecialist daily practice was retained. The selected questions were not modified or edited.

### Development of AmyloGPT

#### Platform and base model

AmyloGPT was developed using GPT Builder (released in November 2023), a no-code platform provided by OpenAI. The underlying model was GPT-4 (the version utilized by GPT Builder as of November 2023). No fine-tuning or supervised training was performed; instead, domain specificity was achieved by uploading a guideline-derived knowledge base and configuring system-level instructions.

#### Knowledge base

The 2020 JCS Guidelines for CA were converted to Microsoft Word format and uploaded as the knowledge base. Word format was chosen over PDF to maximize text-extraction fidelity.

#### System instructions

The following instructions were specified in the system prompt:

1) Role: act as a specialist cardiologist providing expert advice on CA questions posed by nonspecialist physicians in clinical practice.
2) Response strategy: prioritize the uploaded guidelines as the primary reference; use web search functionality to supplement with current information only when the guidelines do not address the question.
3) Information security: do not disclose metadata about uploaded files (filename, format, content details) in responses to minimize information leakage risks.

#### Feature settings

Web search functionality was enabled; image generation (DALL-E) remained at default settings.

#### Response generation protocol

For each question, a new chat session was initiated to prevent context carryover, and the question text alone was input directly, employing zero-shot prompting. No few-shot prompting or additional prompt engineering was used to guide responses. Each question received a single generated response, with no repeat attempts or selective curation of outputs.

**Figure 2:**
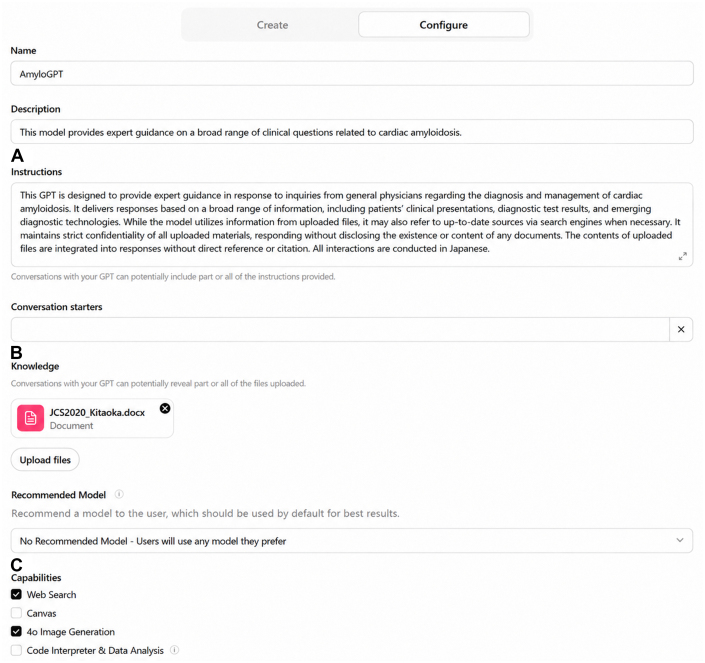
AmyloGPT Configuration Interface Screenshots of GPT Builder settings used to construct AmyloGPT: (A) system instructions (role, response strategy prioritizing the guideline; web search only if unmet), (B) knowledge-base upload (2020 Japanese Circulation Society cardiac amyloidosis guideline, Word format), and (C) feature toggles (web browsing enabled; image generation default). Identifying metadata are masked. This configuration operationalizes a guideline-grounded, no-code retrieval approach without fine-tuning. CA = cardiac amyloidosis; GPT = generative pretrained transformer; JCS = Japanese Circulation Society.

**Figure 3:**
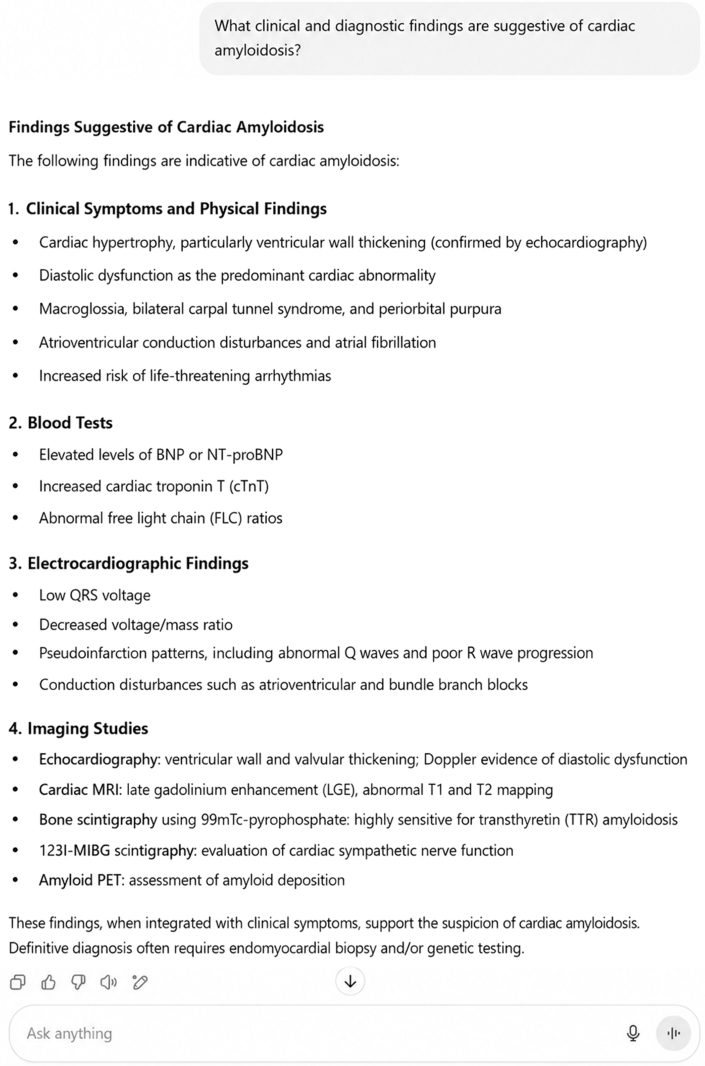
AmyloGPT User Interface Example workflow for generating an answer: a new chat session is initiated, the clinical question is passed verbatim, and a single zero-shot response is produced without repeat attempts or curation. This standardized procedure was applied to all 71 questions to avoid context carryover. BNP = B-type natriuretic peptide; NT-proBNP = N-terminal pro–B-type natriuretic peptide; MIBG = metaiodobenzylguanidine; MRI = magnetic resonance imaging; PET = positron emission tomography.

The overall configuration is summarized in Figure 2. Input/output interface screenshots are provided in Figure 3.

### Response generation by cardiologist answerers

Five cardiologist answerers were randomly assigned 14 to 15 questions each and drafted free-text responses using clinical guidelines, primary literature, and nongenerative online resources, but not generative AI tools.

### Blinding, randomization, and presentation

For each question, paired responses (one from AmyloGPT and one from a board-certified cardiologist answerer) were presented and labeled “Answer A” and “Answer B.” Evaluators were not informed that one response had been generated by AmyloGPT and the other by a board-certified cardiologist answerer. The A/B source assignment was determined by simple randomization with a 1:1 allocation ratio, independently for each question, and was concealed from the evaluators. Nonspecialist evaluators and cardiologist evaluators assessed paired responses to all 71 questions, blinded to the source (AmyloGPT vs cardiologist answers). All questions and evaluations were conducted in Japanese; English translations are provided for reporting purposes. Ratings were collected via standardized Google Forms (Supplemental Figures 1 and 2).

### Outcome measures and assessment

#### Blinded preference

For each pair, evaluators were required to select the superior response (forced choice; ties were not permitted). Based on the randomized allocation, selections were coded as a preference for AmyloGPT or cardiologist answers.

#### Likert ratings

Each response was evaluated on a 5-point Likert scale (1 = very poor, 2 = poor, 3 = neutral, 4 = good, 5 = very good) across prespecified domains.

Nonspecialist evaluators assessed 2 dimensions:

1) Alignment with question intent: the extent to which the response addressed the specific inquiry.
2) Clinical usefulness: the utility of the response for decision-making in clinical practice.

Cardiologist evaluators assessed 5 dimensions:

1) Accuracy: correctness of medical facts and data.
2) Consistency: alignment with guidelines and the current standard of care.
3) Validity: soundness of the clinical reasoning process.
4) Completeness: comprehensiveness of necessary information.
5) Absence of bias: fairness and neutrality (higher scores indicate less bias).

Detailed anchors for each item were provided in the standardized Google Forms (Supplemental Figures 1 and 2).

### Statistical analysis

All statistical analyses were performed using JMP 17 (SAS Institute). A two-sided *P* value of <0.05 was considered statistically significant.

#### Preference data

Preference data from each evaluator-question pair were treated as binomially distributed. We calculated the overall proportion of preference for AmyloGPT along with its Wilson 95% CI. McNemar’s test was used to test the null hypothesis that the preference proportion equals 0.50.

#### Likert scale data

Because Likert scale ratings were ordinal, results were summarized as median (95% CI). For each domain and evaluator group, paired within-question differences between AmyloGPT and cardiologist answers were calculated across the 71 questions and compared against zero using the Wilcoxon signed-rank test (two-sided). We report the median differences with 95% CIs.

#### Supplemental linguistic and structural analysis

We quantified selected linguistic and structural features of the original Japanese responses, including total characters, number of sentences, characters per sentence, and number of bulleted/numbered items. We also recorded whether each response contained any bulleted or numbered list. Continuous or count-based features were summarized as mean (95% CI) and mean differences (95% CI), and paired comparisons were performed using the Wilcoxon signed-rank test. The presence of any bulleted or numbered list was summarized as n (%) and compared using McNemar’s test.

#### Sample size

This was an exploratory study without a formal sample size calculation. The number of questions (n = 71) was determined pragmatically to comprehensively capture the breadth of clinical questions encountered by the 10 nonspecialist physicians.

### Exploratory post hoc analysis of newer therapies

We queried AmyloGPT with 2 additional clinical questions regarding newer therapies (acoramidis and vutrisiran) that were not covered in its primary knowledge base, the 2020 JCS CA guidelines. Web browsing was enabled for these queries. The outputs were reviewed by the investigators for apparent inaccurate or unverified statements. This analysis was conducted outside the prespecified primary comparative evaluation and was not included in the preference or Likert-scale analyses.

## Results

### Characteristics of questions

Of the 106 free- text clinical questions initially submitted by 10 nonspecialist physicians, 71 unique items remained following deduplication and were included in the final analysis. The questions encompassed a broad spectrum of topics, including basic pathophysiology, diagnostic criteria, test selection and interpretation, treatment strategies, patient communication, and health care system–related issues (Figure 1). Representative examples of these questions and the paired responses are shown in Table 1. Supplemental Table 2 presents the full text of 4 selected questions alongside the corresponding responses from both AmyloGPT and cardiologist answerers.

**Table 1:** Representative Questions and Paired Responses

Question: Are There Any Specific Considerations When Monitoring the Clinical Course of Cardiac Amyloidosis at a Primary Care Facility After Initial Treatment at a Specialized Center?	
Respondent	Answer
Cardiologist	Although individual variation exists, patients with cardiac amyloidosis are at increased risk of developing heart failure and arrhythmias over the course of the disease, warranting close attention. The onset of heart failure is associated with a poor prognosis; therefore, routine clinical assessment should include careful evaluation for signs such as dyspnea and peripheral edema. Regarding arrhythmias, patients may experience not only supraventricular arrhythmias such as atrial fibrillation or atrial flutter, but also conduction system disturbances such as atrioventricular block, and ventricular arrhythmias including ventricular tachycardia. It is important to monitor for symptoms such as palpitations, dizziness, or syncope. If such symptoms are present, or if arrhythmias are detected on electrocardiogram, referral to a specialized center is strongly recommended.
AmyloGPT	Key considerations for follow-up care of patients with cardiac amyloidosis in primary settings after initial treatment at a specialized center include the following: 1. Regular cardiac function assessment: Patients with cardiac amyloidosis remain at high risk for progressive cardiac dysfunction and arrhythmias even after treatment. Routine evaluation with echocardiography, electrocardiography, and cardiac MRI is recommended. Serial measurements of biomarkers such as BNP or NT-proBNP are also essential for monitoring cardiac status. 2. Management of arrhythmias: Atrioventricular block and atrial fibrillation occur frequently in cardiac amyloidosis. Any signs of bradyarrhythmia, palpitations, syncope, or presyncope should prompt referral to a cardiologist. The need for pacemaker or implantable cardioverter-defibrillator (ICD) therapy should be assessed as appropriate. 3. Monitoring for heart failure symptoms: Given the progressive nature of heart failure in cardiac amyloidosis, attention should be paid to clinical signs such as peripheral edema, exertional dyspnea, and fatigue. Increases in body weight or the development of ascites may necessitate adjustment of diuretic therapy. 4. Continuation of pharmacotherapy and monitoring for adverse effects: In patients receiving tafamidis or other disease-modifying therapies, long-term medication adherence and monitoring for side effects are critical. Regular evaluation of liver and renal function is advised during ongoing treatment. 5. Management of comorbid conditions: As cardiac amyloidosis is a systemic disorder, attention should also be directed toward renal function, neurological symptoms, and gastrointestinal complications. Renal impairment may affect heart failure management, and periodic laboratory assessments are recommended. 6. Lifestyle counseling and patient education: Guidance on maintaining fluid balance, salt restriction, and appropriate physical activity is essential. Educating patients and their families about disease progression and symptoms that warrant early medical attention is equally important in ensuring optimal long-term outcomes. By addressing these areas, primary care physicians can play a vital role in the longitudinal management of patients with cardiac amyloidosis. Coordination with specialists is encouraged for adjustments in treatment plans as needed.

This table presents representative clinical questions regarding CA generated by nonspecialist physicians, alongside paired responses from AmyloGPT and board-certified cardiologists. All questions and responses were originally generated in Japanese; English translations are provided here for reporting purposes. The content is reproduced verbatim without postediting to authentically reflect the response quality.

BNP = B-type natriuretic peptide; CA = cardiac amyloidosis; MRI = magnetic resonance imaging; NT-proBNP = N-terminal pro–B-type natriuretic peptide.

### Evaluations by nonspecialist physicians

**Figure 4:**
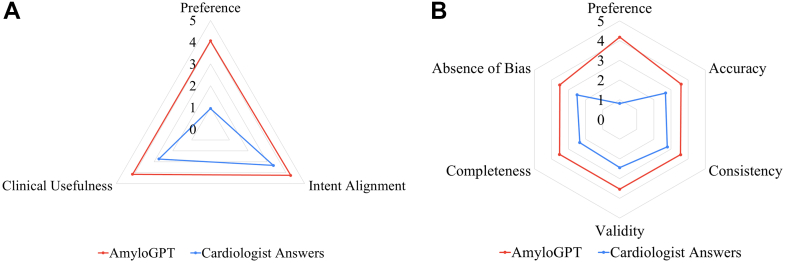
Response Quality by Evaluator Group Radar charts comparing mean ratings for answers provided by AmyloGPT and cardiologists (larger area indicates better performance). Preference rescaled to 0 to 5 for display. (A) Nonspecialist evaluators: preference, intent alignment, and clinical usefulness. Forced-choice preference for AmyloGPT vs cardiologist answers, and mean Likert ratings for intent alignment and clinical usefulness across 71 questions. (B) Cardiologist evaluators: preference, accuracy, consistency, validity, completeness, and absence of bias. Forced-choice preference and mean Likert ratings for accuracy, consistency, validity, completeness, and absence of bias.

Tables 2 and 3 summarize the 710 blinded assessments made by the 10 nonspecialist evaluators (71 questions × 10 evaluators). AmyloGPT was preferred in 81.1% of evaluations (95% CI: 78.1%-83.8%; *P* < 0.001) (Table 2). Regarding the five-point Likert scale ratings, AmyloGPT received significantly higher median ratings than cardiologist answers for alignment with question intent and clinical usefulness (both *P* < 0.001) (Table 3). At the individual evaluator level, the proportion preferring AmyloGPT ranged from 31.0% to 97.2%. A radar plot of preference, intent alignment, and clinical usefulness is shown in Figure 4A.

**Table 2:** Preference for AmyloGPT vs Cardiologist Responses by Evaluator Group

Evaluator Group	Preferred AmyloGPT, % (95% CI)	Preferred Cardiologist Answers, % (95% CI)	*P* Value
Nonspecialist evaluators	81.1 (78.1-83.8)	18.9 (16.2-21.9)	<0.001
Cardiologist evaluators	83.6 (78.6-88.6)	16.4 (11.4-21.4)	<0.001

This table analyzes which response was preferred across 710 evaluations performed by 10 nonspecialist physicians and 213 evaluations performed by 3 board-certified cardiologists. Data are presented as percentages with 95% CIs. *P* values were calculated using McNemar’s test.

**Table 3:** Likert-Scale Quality Ratings by Domain: Nonspecialist and Cardiologist Evaluators

Quality Domain	Nonspecialist Evaluators			*P* Value
	AmyloGPT (95% CI)	Cardiologist Answers (95% CI)	Median Difference (95% CI)	
Intent alignment	5 (4-5)	4 (4-4)	1 (1-1)	<0.001
Clinical usefulness	4 (4-5)	3 (3-3)	1 (1-1)	<0.001

Quality Domain	Cardiologist Evaluators			*P* Value
	AmyloGPT (95% CI)	Cardiologist Answers (95% CI)	Median Difference (95% CI)	
Accuracy	4 (4-4)	3 (3-3)	1 (1-1)	<0.001
Consistency	4 (4-4)	3 (3-3)	1 (0-1)	<0.001
Validity	4 (4-4)	3 (3-3)	1 (1-1)	<0.001
Completeness	4 (4-4)	3 (3-3)	1 (1-1)	<0.001
Absence of bias	4 (4-4)	3 (3-3)	1 (1-1)	<0.001

Summary of median Likert scale scores (1 = very poor to 5 = very good) assessed by 10 nonspecialist evaluators (n = 710 evaluations) and 3 cardiologist evaluators (n = 213 evaluations). Data are presented as the median score for each group and the median difference (AmyloGPT – Cardiologist answers), both with 95% CIs. *P* values were calculated using the Wilcoxon signed-rank test.

**Central Illustration:**
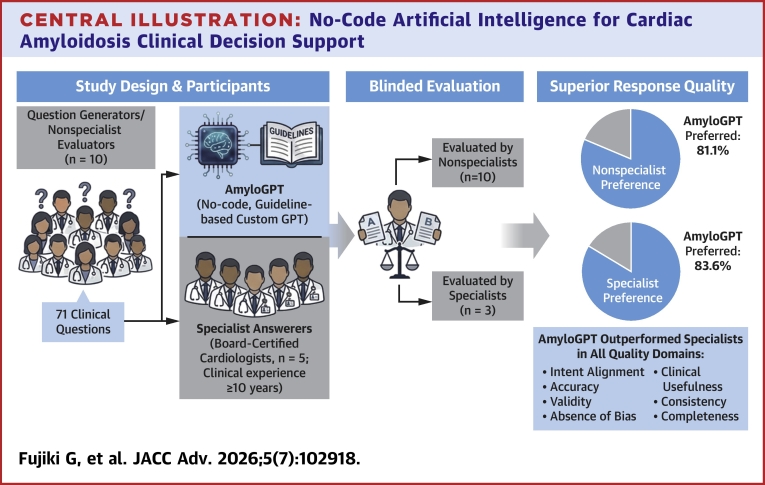
No-Code Artificial Intelligence for Cardiac Amyloidosis Clinical Decision Support Overview of the study design and key findings. AmyloGPT, a guideline-based, no-code custom generative pretrained transformer built from the 2020 Japanese Circulation Society cardiac amyloidosis guideline, was evaluated in a blinded head-to-head comparison against cardiologist answerers using 71 clinical questions. Question generators/nonspecialist evaluators (n = 10) and cardiologist evaluators (n = 3) rated paired responses; AmyloGPT was preferred in 81.1% and 83.6% of evaluations, respectively (both *P* < 0.001), and achieved higher Likert ratings across quality domains. This accessible, guideline-grounded large language model may help shorten diagnostic delays and reduce geographic inequalities in cardiac amyloidosis care. GPT = generative pretrained transformer.

### Evaluations by cardiologist evaluators

Tables 2 and 3 summarize the 213 blinded assessments made by the 3 cardiologist evaluators (71 questions × 3 evaluators). AmyloGPT was preferred in 83.6% of evaluations (95% CI: 78.6%-88.6%; *P* < 0.001) (Table 2). Regarding the five-point Likert scale ratings, AmyloGPT received significantly higher median ratings than cardiologist answers across all 5 domains: accuracy, consistency, validity, completeness, and absence of bias (all *P* < 0.001) (Table 3). At the individual evaluator level, the proportion of preference for AmyloGPT ranged from 83.1% to 84.5%. A radar chart visualizing the preference rates and scores across all 5 quality dimensions is presented in Figure 4B.

### Exploratory post hoc analysis of newer therapies

In the exploratory post hoc analysis of newer therapies, AmyloGPT generated responses that included several inaccurate or unverified statements. Specifically, the model incorrectly cited a clinical trial name and provided outdated information regarding current regulatory approvals. These results are shown in Table 4.

**Table 4:** AmyloGPT Responses to Questions Regarding Newer Therapies

Question	Is Acoramidis (Beyonttra) Effective for Cardiac Amyloidosis?
AmyloGPT response	Acoramidis (brand name: Beyonttra) is a promising therapeutic agent for transthyretin-type cardiac amyloidosis (ATTR-CM). Its efficacy is supported by clinical data, particularly from the ATTR-ACT trial, a global phase 3 study. In this trial, acoramidis significantly reduced cardiovascular mortality and heart failure-related hospitalizations compared to placebo. It also improved health-related quality of life, as measured by the Kansas City Cardiomyopathy Questionnaire-overall Summary (KCCQ-OS) score. The efficacy was especially pronounced in patients with wild-type ATTR-CM and also showed benefit in those with hereditary forms. Therefore, acoramidis (Beyonttra) is considered effective in treating cardiac amyloidosis, particularly ATTR-CM. However, in Japan, it's essential to confirm its approved indications and insurance coverage, and its use should be tailored to the specific subtype of cardiac amyloidosis.

Question	What Kind of Drug Is Vutrisiran (Amvuttra)?
AmyloGPT response	Vutrisiran (brand name: Amvuttra) is a treatment for hereditary transthyretin (TTR) amyloidosis (hATTR amyloidosis). It is classified as a nucleic acid-based drug known as small interfering RNA (siRNA), which works by degrading TTR mRNA in the liver and thereby suppressing the synthesis of TTR protein. Like the earlier siRNA drug patisiran (Onpattro), vutrisiran aims to reduce TTR production, but it has several distinct features: It is administered subcutaneously once every 4 weeks, offering greater convenience. It is conjugated with GalNAc (N-acetylgalactosamine), which enhances targeted delivery to the liver. In clinical trials (such as the HELIOS-A study), it has shown improvements in cardiac function and quality of life, suggesting potential benefit in managing cardiac amyloidosis symptoms. Currently, vutrisiran is approved primarily for patients with hATTR amyloidosis who present with polyneuropathy symptoms, but its benefits for cardiac involvement are also being recognized, and future expansion of its indications is anticipated. Let me know if you'd like a summary of the HELIOS-A trial or comparisons with other treatments.

This table provides verbatim AmyloGPT outputs to 2 post hoc questions regarding newer therapies (acoramidis and vutrisiran) that were not covered in the 2020 JCS CA guideline knowledge base. These examples illustrate the risk of hallucinations; in the acoramidis example, the ATTRibute-CM trial was incorrectly cited as ATTR-ACT, and in the vutrisiran example, the output implied that ATTR-CM is not currently an approved indication, although labeling includes ATTR-CM in some regions.

ATTR-ACT = Transthyretin Amyloidosis Cardiomyopathy Clinical Trial; ATTR-CM = transthyretin amyloid cardiomyopathy; ATTRibute-CM = Efficacy and Safety of AG10 in Subjects with Transthyretin Amyloid Cardiomyopathy; CA = cardiac amyloidosis; HELIOS-A = phase 3 study of vutrisiran in hereditary transthyretin amyloidosis; JCS = Japanese Circulation Society.

### Supplemental linguistic and structural analysis

Analysis of the original Japanese responses showed that AmyloGPT responses were not only substantially longer than cardiologist responses but also contained more sentences and more bulleted/numbered items (Supplemental Table 3). The mean characters per sentence did not differ significantly between groups. In addition, bulleted or numbered lists were present in 60 of 71 AmyloGPT responses (84.5%) compared with 3 of 71 cardiologist responses (4.2%; *P* < 0.001).

## Discussion

In this prospective, blinded, head-to-head comparative study, we evaluated the response quality of a no-code, guideline-based custom GPT (AmyloGPT) against board-certified cardiologists using 71 clinical questions. Across 710 assessments by nonspecialist evaluators (n = 10) and 213 assessments by cardiologist evaluators (n = 3), AmyloGPT was preferred in 81.1% (95% CI: 78.1%-83.8%) and 83.6% (95% CI: 78.6%-88.6%) of evaluations, respectively (both *P* < 0.001). Regarding the five-point Likert scale ratings, AmyloGPT received significantly higher median ratings for alignment with question intent and clinical usefulness among nonspecialists, and for accuracy, consistency, validity, completeness, and absence of bias among cardiologist evaluators (all *P* < 0.001).

The novelty of this study lies in applying a no-code, guideline-based custom GPT to CA—a rare disease with substantial diagnostic complexity. Although the internal architecture of GPT Builder is undisclosed, its behavior is functionally analogous to retrieval-augmented generation, in which relevant passages from an external knowledge base are used to condition the output. This approach has been reported to enhance the reliability of generated content.^14^ Such no-code platforms may advance the democratization of medical AI development by allowing clinicians to build and update disease-specific systems without reliance on external engineers. This generalizable approach may also apply to other rare diseases with consensus-based guidelines.

Our findings are consistent with prior work demonstrating that domain-specific LLMs can match or exceed specialist clinical judgment.^15^^,^^16^ AmyloGPT also consistently provided more structured responses, which may have influenced evaluator preferences.^17^ Supplemental analyses showed that, compared with cardiologist responses, AmyloGPT responses were longer, contained more sentences, and more frequently used bulleted or numbered items, whereas mean characters per sentence did not differ significantly between groups (Supplemental Table 3). These findings suggest that the observed preference for AmyloGPT may have reflected not only substantive differences in response quality but also differences in presentation style.

From a clinical perspective, this study suggests a pragmatic pathway toward earlier diagnosis and treatment in CA. In transthyretin amyloid cardiomyopathy, diagnostic delays average 34 months,^18^ patients undergo a median of 17 health care visits,^19^ and 33% consult ≥5 health care professionals before the correct diagnosis.^20^ Longer diagnostic delay in wild-type transthyretin amyloid cardiomyopathy is associated with higher all-cause mortality,^21^ and earlier tafamidis initiation improves survival.^22^ By providing immediate, guideline-based answers to practical questions, AmyloGPT may help clinicians recognize red-flag features earlier and facilitate referral, particularly in community hospitals and rural settings where specialist access is limited. However, whether its use in routine practice improves diagnosis, referral, testing, or treatment initiation remains unknown and should be evaluated in prospective implementation studies.

### Study limitations

This study has several limitations. First, the questions were generated by nonspecialists and may not fully reflect routine care queries. In addition, this study did not assess real-world impact on patient management; prospective implementation studies are warranted. Second, the cardiologist responses were drafted by 5 individual board-certified cardiologists and may not fully reflect the broader community consensus. Third, AmyloGPT's knowledge base is based on the 2020 JCS CA guideline. Our post hoc analysis revealed inaccurate statements regarding newer therapies, highlighting the need for systematic content updates and verification against primary sources. Fourth, LLMs can generate hallucinations (reported in a prior study at 1.47%).^23^ As this study focused on comparative preference and Likert-rated response quality, we did not systematically evaluate potentially harmful outputs such as hallucinations or dangerous medical advice. Future studies should incorporate such assessments, and physician oversight remains essential. Fifth, although responses were presented as blinded A/B pairs, we cannot exclude the possibility that evaluators inferred that one of the responses was AI-generated based on stylistic or structural features. Sixth, the evaluation was only in Japanese; multilingual performance and regional adaptability were not examined. Finally, as LLM capabilities rapidly evolve, the findings of this study may not fully reflect the performance of newer models; validating them with recent models is an important future step.

## Conclusions

In this prospective, blinded, head-to-head comparative study, a no-code, guideline-based custom GPT (AmyloGPT) delivered better response quality than that of board-certified cardiologists for clinical questions regarding CA. By demonstrating feasibility on a no-code platform, we show that clinicians without programming expertise can build and deploy effective, disease-specific LLMs. In health care systems constrained by specialist shortages and geographic inequalities, AI-enabled knowledge dissemination may become an important component of equitable, evidence-based care. However, further studies are needed to evaluate potentially inaccurate outputs such as hallucinations.

Perspectives **COMPETENCY IN MEDICAL KNOWLEDGE:** A no-code, guideline-based custom generative pretrained transformer (AmyloGPT) demonstrated response quality superior to that of board-certified cardiologists in a blinded evaluation of answers to clinical questions regarding cardiac amyloidosis. These findings support the view that systematically grounding large language model outputs in authoritative clinical guidelines may help improve accuracy and clinical utility, even in complex, rare-disease domains where specialist expertise is limited.

**TRANSLATIONAL OUTLOOK:** The feasibility of developing high-performance, disease-specific artificial intelligence tools without programming expertise offers a scalable and pragmatic pathway to democratize specialized medical knowledge. Integrating such accessible tools into routine clinical workflows could bridge the gap between nonspecialist and expert care, particularly in primary care and community hospital settings, potentially shortening diagnostic delays and mitigating geographic disparities in patient access to evidence-based management of rare diseases like cardiac amyloidosis.

## Funding support and author disclosures

This study was supported by the Cross-ministerial Strategic Innovation Promotion Program (SIP) on “Integrated Health Care System” (Grant Number: JPJ012425). Dr Kodera has received research grant support from 10.13039/100018504Nipro Corporation. All other authors have reported that they have no relationships relevant to the contents of this paper to disclose.
