# Supplementary material for: A No-Code, Guideline-Based Custom GPT Outperforms Cardiologists in Response Quality for Cardiac Amyloidosis
Source: JACC Adv. 2026 Jul 22;5(7):102918. doi: 10.1016/j.jacadv.2026.102918 (PMC13400095; doi:10.1016/j.jacadv.2026.102918)
Supplement: Supplemental Tables and Figures [file mmc1.docx]

**Supplemental Table 1. Participant Demographics and Professional Background**

(A) Question Generators / Non-specialist Evaluators

| Physician | Institution Type | Specialty | Clinical Experience  (year) |
| --- | --- | --- | --- |
| 1 | Municipal Acute-Care Hospital | Gastroenterology | 4 |
| 2 | University Hospital | Cardiology | 3 |
| 3 | Private Hospital | Gastroenterology | 12 |
| 4 | University Hospital | Rheumatology (Collagen Disease) | 12 |
| 5 | University Hospital | Breast surgery | 12 |
| 6 | Clinic | Gastroenterology | 14 |
| 7 | University Hospital | Cardiology | 3 |
| 8 | University Hospital | Cardiology | 3 |
| 9 | University Hospital | Cardiology | 4 |
| 10 | University Hospital | Cardiology | 4 |

(B) Cardiologist Answerers (board-certified cardiologists)

| Physician | Institution Type | Clinical Experience  (year) |
| --- | --- | --- |
| 1 | Private Hospital | 11 |
| 2 | Clinic | 18 |
| 3 | Acute-Care Hospital | 14 |
| 4 | University Hospital | 13 |
| 5 | Acute-Care Hospital | 14 |

(C) Cardiologist Evaluators (board-certified cardiologists)

| Physician | Institution Type | Subspecialty | Clinical Experience  (year) |
| --- | --- | --- | --- |
| 1 | University Hospital | Heart Failure | 17 |
| 2 | University Hospital | Arrhythmia | 21 |
| 3 | University Hospital | Critical Care | 12 |

Participants are grouped as (A) question generators / non-specialist evaluators (n = 10), (B) cardiologist answerers (board-certified; n = 5), and (C) cardiologist evaluators (board-certified; n = 3). Reported variables include institution type, specialty/subspecialty, and years of clinical experience.

**Supplemental Table 2. Selected Dataset of Four Questions with Paired Responses**

(A) Question. Please advise on the appropriate timing for referring patients with cardiac amyloidosis to a specialist.

| Respondent | Answer |
| --- | --- |
| Cardiologist | This applies when, in addition to heart failure symptoms, red-flag symptoms/findings are present (family history; autonomic dysfunction; cardiac findings such as low voltage despite left ventricular hypertrophy or atrioventricular block; carpal tunnel syndrome; gastrointestinal symptoms; ocular symptoms; unexplained weight loss; and resistance to immunotherapy, among others). |
| Amylo  GPT | Referral to a specialist for a patient with cardiac amyloidosis should be considered in the following situations:  1. When cardiac amyloidosis is suspected based on clinical symptoms or test findings  If any of the following are present, cardiac amyloidosis should be suspected and referral to a specialist is recommended:   - Heart failure of unknown cause, particularly heart failure with preserved left ventricular ejection fraction (HFpEF). - Left ventricular hypertrophy without an identifiable cause such as hypertension. - Carpal tunnel syndrome, especially when bilateral. - Arrhythmias (e.g., atrial fibrillation) or conduction abnormalities (e.g., atrioventricular block). - Laboratory or imaging findings suggestive of amyloidosis (e.g., disproportionately elevated NT-proBNP or troponin, abnormal findings on echocardiography).   2. When diagnostic testing is required to establish a definitive diagnosis  Patients who are considered to require confirmatory testing—such as endomyocardial biopsy or nuclear imaging (e.g., technetium-99m pyrophosphate scintigraphy)—should be referred to a specialist.  3. When hereditary amyloidosis is suspected  In particular, when ATTRv (hereditary transthyretin amyloidosis) is suspected, referral is advisable because genetic testing and genetic counseling may be necessary.  4. When treatment decisions are complex or uncertain  Specialist input is recommended when determining indications for disease-modifying therapies (e.g., tafamidis) and establishing an appropriate treatment strategy.  If these conditions are met, consider early referral to a specialist experienced in the diagnosis and management of cardiac amyloidosis to facilitate timely diagnosis and treatment. |

(B) Question. Where is the specialized hospital for cardiac amyloidosis?

| Respondent | Answer |
| --- | --- |
| Cardiologist | A multidisciplinary diagnostic workup—including cardiac MRI, scintigraphy, and endomyocardial biopsy—is required to establish the diagnosis of cardiac amyloidosis and to initiate treatment; therefore, referral to specialized centers such as regional university hospitals or the National Cerebral and Cardiovascular Center is considered desirable. Treating institutions can also be identified through online searches, and region-specific hospitals can be searched via the following website:  https://www.janssenamyloidosis.jp/ |
| Amylo  GPT | Specialized hospitals and medical institutions for cardiac amyloidosis include the following:  1. National Cerebral and Cardiovascular Center (Osaka)  The Department of Cardiovascular Medicine—particularly the Heart Failure Division—provides diagnosis and treatment for cardiac amyloidosis.  2. Kochi University Medical School (Kochi)  The Division of Geriatric Medicine and Cardiovascular Medicine is engaged in both clinical care and research on cardiac amyloidosis.  3. Kurume University School of Medicine (Fukuoka)  The Department of Cardiology and Vascular Medicine is involved in the diagnosis and treatment of cardiac amyloidosis.  4. Kita-Alps Medical Center Azumi Hospital (Nagano)  Provides specialized care focused on cardiac amyloidosis.  5. Keio University School of Medicine (Tokyo)  The Department of Cardiology serves as a key center for the management of cardiac amyloidosis.  These institutions have also been involved in developing clinical practice guidelines for cardiac amyloidosis and provide specialized care, with experienced physicians and established diagnostic and treatment systems. |

(C) Question. Which patients are eligible for tafamidis treatment for cardiac amyloidosis?

| Respondent | Answer |
| --- | --- |
| Cardiologist | The patient has heart failure, an interventricular septal thickness of ≥12 mm, amyloid deposition confirmed on biopsy, and identification of the TTR precursor protein by immunohistochemistry. |
| Amylo  GPT | Patients with cardiac amyloidosis who are eligible for tafamidis (tafamidis formulations) are primarily those with transthyretin cardiac amyloidosis (ATTR amyloidosis). More specifically, the following patient populations are considered candidates:  1. Wild-type transthyretin cardiac amyloidosis (ATTRwt)  ATTRwt is caused by deposition of wild-type (non-mutant) transthyretin and predominantly affects the heart, leading to heart failure and left ventricular hypertrophy.  2. Hereditary transthyretin amyloidosis (ATTRv)  ATTRv is caused by mutant transthyretin that forms amyloid deposits, affecting the heart and/or peripheral nerves. Tafamidis is also indicated in some patients who have concomitant neuropathy.  When initiating tafamidis, the following factors are generally taken into account:   - A confirmed diagnosis: Tafamidis is generally recommended only for patients with a confirmed diagnosis of cardiac amyloidosis. - Presence of cardiac involvement and symptoms: Patients typically have echocardiographic evidence and/or clinical symptoms consistent with cardiac involvement, such as heart failure.   Use of tafamidis should be considered in cases deemed appropriate by physicians experienced in the diagnosis and management of cardiac amyloidosis. |

(D) Question. Please provide the diagnostic criteria for cardiac amyloidosis.

| Respondent | Answer |
| --- | --- |
| Cardiologist | The diagnostic criteria for cardiac amyloidosis are as follows.  Items A, B, and C are common to all types of cardiac amyloidosis, while the remaining items differ by subtype.  A. Presence of clinical symptoms or test findings considered attributable to amyloidosis.  B. Tissue biopsy shows amyloid deposition that is Congo red–positive and demonstrates apple-green birefringence under polarized light microscopy.  C. The amyloid deposits are positive for the precursor protein specific to each subtype.  AL amyloidosis  D. An M-protein is detected in blood and/or urine.  E. Other diseases that could cause similar clinical symptoms or test findings are adequately excluded.  Definite: ≥1 item of A, and B, C, D, and E are fulfilled; or  ≥2 items of A, and B, C, and E are fulfilled.  Probable: ≥1 item of A, and B, D, and E are fulfilled.  Wild-type ATTR (ATTRwt)  D. Positive cardiac uptake is confirmed on 99mTc-pyrophosphate scintigraphy.  E. No TTR gene mutation that causes an amino acid substitution is identified.  F. No M-protein is detected.  G1. Exclude localized amyloidosis confined only to tendon/ligament tissue.  G2. Other diseases that could cause similar clinical symptoms or test findings are adequately excluded.  Definite: A, B, C, E, and G1 are fulfilled.  Probable: A, D, E, F, and G2 are fulfilled.  Hereditary ATTR (ATTRv)  D. Positive cardiac uptake is confirmed on 99mTc-pyrophosphate scintigraphy.  E. ATTR gene mutation that causes an amino acid substitution is identified.  Definite: A, B, C, and E are fulfilled.  Probable: A, B, and E are fulfilled, or A, D, and E are fulfilled. |
| Amylo  GPT | The diagnostic criteria for cardiac amyloidosis are defined as follows:  1. Clinical symptoms and test findings that raise suspicion for cardiac amyloidosis   - Clinical manifestations include heart failure, conduction disturbances such as atrioventricular block or bundle branch block, atrial fibrillation, macroglossia, and carpal tunnel syndrome. - Cardiac amyloidosis should be suspected when echocardiography or electrocardiography demonstrates findings such as low voltage, abnormal Q waves, or a pseudo-infarction pattern.   2. Common diagnostic components across subtypes   - Confirmation of amyloid fibrils on endomyocardial biopsy or abdominal fat pad biopsy. - Identification of characteristic findings on imaging studies such as cardiac MRI, bone scintigraphy, or amyloid PET.   3. Diagnostic criteria for AL amyloidosis   - Evidence of elevated monoclonal immunoglobulin light chains (free light chains, FLC) due to plasma cell dyscrasia. - Demonstration of plasma cell abnormalities on bone marrow aspiration and/or biopsy.   4. Diagnostic criteria for ATTR (transthyretin) amyloidosis   - Assessment of cardiac uptake on technetium-99m pyrophosphate scintigraphy. - For hereditary ATTR amyloidosis (ATTRv), confirmation of a TTR mutation by genetic testing. - For wild-type (non-hereditary) ATTR amyloidosis (ATTRwt), an integrated assessment incorporating the clinical history and clinical findings.   Based on these criteria, the diagnosis of cardiac amyloidosis is established through a stepwise process from clinical suspicion to imaging evaluation and, when necessary, pathological confirmation by biopsy. For detailed diagnostic algorithms, consultation of the JCS guidelines is recommended. |

Four representative question–response pairs illustrating observed preference patterns: (A) both non-specialist and cardiologist evaluators preferred AmyloGPT; (B) non-specialist evaluators preferred AmyloGPT while cardiologist evaluators preferred the cardiologist response; (C) non-specialist evaluators showed no majority preference (5–5 split), whereas cardiologist evaluators preferred AmyloGPT; and (D) both groups preferred the cardiologist response. No question showed a non-specialist majority for the cardiologist response.

**Supplemental Table 3. Linguistic and Structural Features of the Original Japanese Responses from AmyloGPT and Cardiologists**

|  | AmyloGPT (95% CI) | Cardiologist Answers (95% CI) | Mean Difference (95% CI) | P Value |
| --- | --- | --- | --- | --- |
| Characters | 613 (563 - 663) | 115 (89 – 141) | 498 (445 – 550) | <0.001 |
| Sentences | 13 (12 – 15) | 2 (2 – 2) | 11 (10 – 13) | <0.001 |
| Characters per sentence | 50 (46 – 54) | 57 (49 – 65) | 7 (-1 – 15) | 0.271 |
| Number of bulleted/numbered items | 6 (5 – 7) | 0 (0 – 1) | 6 (5 – 7) | <0.001 |
| Responses containing any bulleted/numbered list, n (%) | 60 / 71 (84.5%) | 3 / 71 (4.2%) | – | <0.001 |

Values are presented as mean (95% CI), unless otherwise indicated. P values for continuous or count-based features were calculated using the Wilcoxon signed-rank test. The presence of any bulleted or numbered list was presented as n (%) and compared using McNemar’s test.

CI = confidence interval.

**SUPPLEMENTAL FIGURE 1 Evaluation Questionnaire for Non-specialist Evaluators**


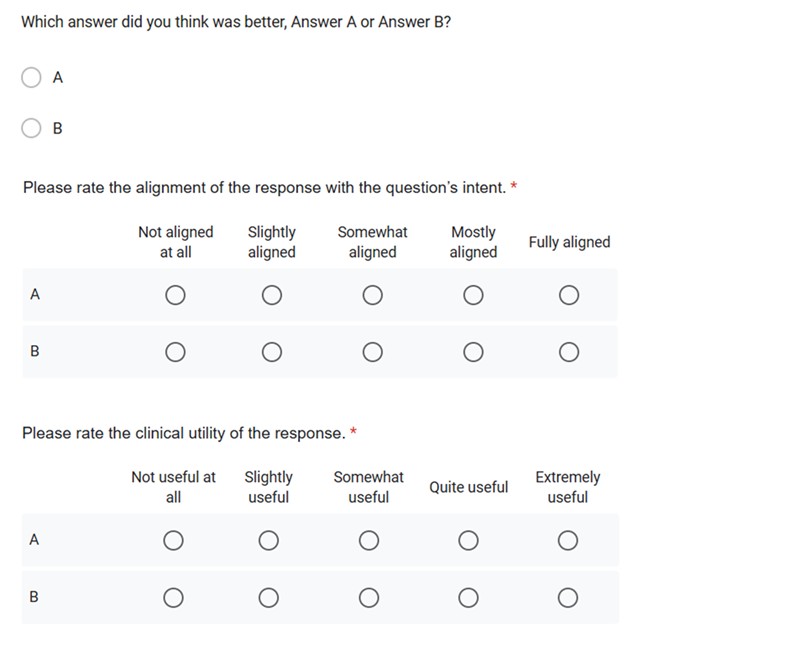


Screenshot of the Google Form used by non-specialists. The question is shown with two anonymized responses (“Answer A” / “Answer B”), randomized 1:1 and blinded. Evaluators recorded a forced-choice preference and scored intent alignment and clinical usefulness on 5-point Likert scales.

**SUPPLEMENTAL FIGURE 2 Evaluation Questionnaire for Cardiologist Evaluators**


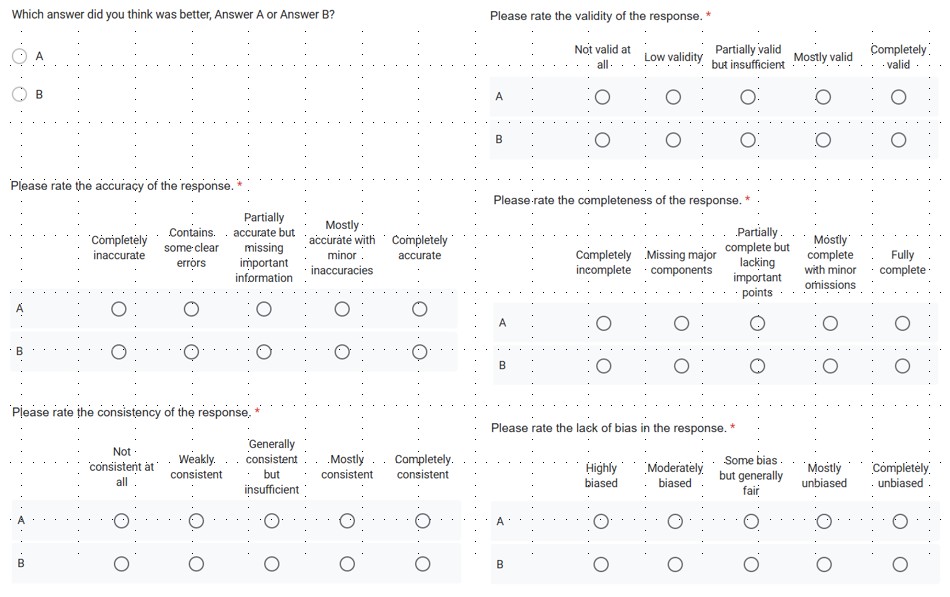


Screenshot of the Google Form used by cardiologist evaluators. The question is shown with two anonymized responses (“Answer A” / “Answer B”), randomized 1:1 and blinded. Evaluators chose a forced-choice preference and rated accuracy, consistency, validity, completeness, and absence of bias on 5-point Likert scales.
